# Editorial

**Published:** 1880-07

**Authors:** 


### EDITORIAL DEPARTMENT.

### SOME LEGISLATIVE DOINGS—FAILURE OF THE VETERINARY BILL, ETC.

We congratulate veterinarians, and the public in general, that the bill to regulate the practice of veterinary medicine and surgery, brought up in our State Legislature at its last session, failed to pass. We are glad to say, too, that the anti-vivisection bill, which, at the time of its discussion, excited such a widespread interest, was also defeated. The bill apprporiating $35,000 for the prevention of pleuro-pneumonia passed, and is a measure which meets our hearty concurrence.

---

The report of the United States Commissioner of Agriculture for the year 1878, has lately been received, and is, as usual, full of highly interesting and practical information for all who are engaged in agricultural pursuits. It exhibits in a concise and comprehensive form the results achieved by the labor and capital of that portion of our population which is engaged in the various branches of husbandry, and indicates other directions in which these may be still further employed with as great, if not, indeed, a greater, degree of success. The elaborate statistics tabulated show at a glance the degree in which each and all of our great and varied productive interests are prospering, and makes comparisons between our own nation and others competing in the same branches. There seems, indeed, to be scarcely anything of practical importance in all our varied industries that has escaped notice. The animal, vegetable and mineral kingdoms get each its due share of attention, and every separate commonwealth of our wide and extended domain has its representative. It treats of plants and animals which in health not only yield to the cultivator and stock raiser their resources of wealth and pleasure, but it treats also of the enemies and diseases by which these are liable to be attacked, such as insects and worms which blight and destroy, and the plagues and pestilence which move in darkness and silence to the work of desolation. We would call the attention of our readers to the veterinary department, and especially to the reports on the swine plague of Drs. Detmer and Law; and we would recommend all to avail themselves of such opportunities as may offer to examine these, for they are full of very important practical facts, developed by well-planned and efficiently executed experiments, supplemented by all the assistance which the sciences of chemistry and microscopy could render. These experiments have now settled conclusively the question for so many years under discussion as to whether the *hog plague*, erroneously called *hog cholera*, is or is not a highly infectious and contagious disease, by an affirmative answer. They have developed the fact, that there exists in all parts of the infected animal, and especially in the fluids a specific germ by means of which the disease is propagated as surely as smallpox reproduces itself in the human system; that these germs may be preserved in the tissues in a dried state for weeks, and in some cases even for months, and that no amount of cold will destroy them. They do not inform us whether heat will destroy them or not, but state that decomposition or putrefaction will destroy their vitality, and that the infection may be communicated from one herd to another through the agency of rats and other small animals, which may themselves contract the disease from eating the dejections of infected animals.

In order to stay the progress of this plague, and, if possible, to eradicate the contagion entirely, it is recommended that the diseased animals be slaughtered, and their carcases buried in the ground, at least four feet below the surface. The first part of this recommendation we heartily endorse. Let the infected animals be destroyed by all means, but as to the disposition of the carcases in the way proposed, that is something to which we decidedly object. In this case, the final destruction of the disease-producing germ could take place only through decomposition—

putrefaction. Buried at this depth in the ground, decomposition must necessarily go on very slowly. In the meantime they are accessible to the approaches of dogs and other animals which might unearth them; also to rats, mice, and other rodents, which might burrow down to them, themselves thus contract the disease, and afterwards convey it to distant herds. Again, this method is objectionable on account of the danger of spreading the infection by the pollution of springs and wells in the vicinity of the buried animals. As the disease-producing germs are effectually destroyed by decomposition, we would advise that the carcases of infected animals be made, with other materials into a compost, and thus converted into fertilizers. Another plan suggested for their destruction is *cremation*—burning the carcase. This, too, we would favor.

We take this opportunity of congratulating the country and of complimenting Congress upon the making of an appropriation to inaugurate and carry to a successful issue, an investigation which has resulted so fortunately in the disclosure of the true nature of this epidemic among swine, and the discovery of the existence of the specific germ, the *bacillus suis*, to the presence of which the disease is alone due; such presence, when demonstrable—which in most cases it is at a very early period of the disease—rendering the diagnosis unquestionable. The report on the contagion also details the methods of its advance When the results achieved are compared with the extent of the appropriations made for the purpose, we are free to say that never before in this country has there been so great a return for so small an outlay; never before has the country received so much benefit from the expenditure of so small an amount of money. The fund placed by Congress at the disposal of the Commissioner of Agriculture to pay the expenses of a commission to investigate the most contagious and destructive of the diseases to which our domestic animals are liable, to discover, if possible, their nature and causes, and to suggest the remedy, was $10,000, the greater portion of which was applied to the investigation of the prevailing epidemic among swine; as that was a disease which had, in the year previous, destroyed nearly $2,000,000 worth of property in swine, and which, in the year immediately succeeding, would doubtless have destroyed more than double that amount in this species of live stock, had the epidemic been allowed to continue unchecked by the inauguration of measures based upon the results obtained and suggested by the knowledge acquired from the investigation had, assisted and sustained by appropriate legislation.

Our Congress seems to be as unwilling to appropriate money to discover and destroy *bacteria* and insects, as it is to furnish the sinews of war with which to fight the Indians; but is not at all afraid of contractors, mud-diggers, etc.

We venture to say that had our government, to arrest diseases prevailing epidemically among our flocks and herds, and the progress of the armies of insects invading the growing crops of the husbandmen—through both of which agencies values to the amount of millions upon millions are destroyed annually, and the capital which these represent totally annihilated—expended one quarter of the sum it now spends in dredging rivers unnavigable by nature, and harbours without commerce, it would have added to the wealth of our common country, tenfold the amount to be gained by any such expedients as the last-named—expedients which are doubtful at best, and further, objectionable, as partaking too much of the character of special legislation, as contrasted with that which has for its object the general welfare of the whole country; thereby showing in our legislators a lack of wisdom which we trust these representatives of the people will not again exhibit.

---

An article, entitled "Does Vivisection Pay?" published in a late number of *Scribner's Monthly*, demands a passing notice at our hands, as it is calculated to effect, in some degree, physiological inquiry in the future. We do not purpose to enter into any lengthy discussion of this paper, as it is rather of a sentimental character, to which no substantial argument can be opposed. We shall therefore merely notice some points made by the writer, and which we conceive to be not well taken, and briefly criticise some of his proposed methods.

In the first place, we must take exception to the writer's estimate of the spirit and meaning of those who, in this branch of science, are engaged in the pursuit of experimental knowledge, and who are eager to communicate in the same manner the knowledge thus acquired to those who are, in the near future, to become their colleagues and successors. He would lead the public—if we rightly interpret his sentiment—to regard our Daltons, our Flints, our Arnolds, and others engaged in the pursuit of physiological knowledge for the benefit of suffering humanity, by means of experiments made upon the lower orders of creation, as men who delight in the sufferings caused by such experiments, and the experiments themselves worse than useless. The writer might fancy that a knowledge of surgery sufficient for all practical purposes can be obtained by the study of treatises on surgical practice, of which there is an abundance; but would he undertake the ligature of the femoral or the carotid artery, with full reliance upon his knowledge of the anatomy of the parts thus acquired, without first having witnessed the operation as performed by a competent surgeon, if not upon a human subject—for such are not always available at the proper time—then at least upon one of the lower animals?

In order to prevent abuses, the writer of this article would make vivisection the subject of legislative enactment. He would have officers appointed whose function it should be to see that no unnecessary suffering should be inflicted upon the brute creation by the lords thereof in order to gratify their insatiable thirst for knowledge. Regarding the manner in which, under our political system, appointments to office are usually made, it might happen that this would be to place over the heads of these scholarly gentlemen and learned professors, some local politician who had received his appointment in reward for partisan services; perhaps some Fourth Ward rough, who had been given the office in consideration of the *knock-down* arguments advanced by him at some primary election in his district; either of whom would make of the position, only a stepping stone to further advancement to a higher office with better pay; the chief and perhaps only qualification in either case being the ability to draw their salaries.

It is an old maxim that "That state is governed best which is governed least." In our humble opinion, matters of this kind are best left alone, without any legal restrictions whatsoever, and thus allowed to regulate themselves. From the character and standing of the men whom usually we find engaged in scientific pursuits, especially those of the medical profession, we have, for our part, no fears for the result. Nevertheless, it is well enough to discuss the matter in all its bearings, regulate it by public opinion, if you can. This, after all, in a free government, with a free press, is the court of last resort. But we deprecate any interference in the matter by the legislature, at least at present.

