# Errata

**Published:** 1880-07

**Authors:** 


ERRATA.—On page 160 top line, instead of "nearly $2,000,000," read "$12,000,000 worth of property."

